# Self-Reported Alcohol Abuse and the Desire to Receive Mental Health Counselling Predict Suicidal Thoughts/Thoughts of Self-Harm among Female Residents of Fort McMurray

**DOI:** 10.3390/ijerph192013620

**Published:** 2022-10-20

**Authors:** Belinda Agyapong, Reham Shalaby, Ejemai Eboreime, Yifeng Wei, Vincent I. O. Agyapong

**Affiliations:** 1 Department of Psychiatry, University of Alberta, Edmonton, AB T6G 2B7, Canada; 2 Department of Psychiatry, Dalhousie University, Halifax, NS B3H 2E2, Canada

**Keywords:** multiple traumas, suicidal ideation, mental health, Fort McMurray, female

## Abstract

Suicidal ideation and thoughts of self-harm continue to be challenging public health problems. It is presently unknown what the prevalence and correlates of suicidal thoughts and self-harm are in female residents of Fort McMurray, a city that has endured wildfires, flooding, and the COVID-19 pandemic in the last five years. This study aimed to determine the prevalence and correlates of suicidal ideation and thoughts of self-harm among female residents of Fort McMurray. A cross-sectional study using an online survey questionnaire was used to collect sociodemographic and clinical information from the residents of Fort McMurray between 24 April and 2 June 2021. Suicidal ideation and thoughts of self-harm among females were assessed using the ninth question of the Patient Health Questionnaire-9, a validated screening tool used to assess depression symptoms. Likely generalized anxiety disorder (GAD), major depressive disorder (MDD), post-traumatic stress disorder (PTSD) and low resilience were measured using standardized rating scales. Data were analyzed with SPSS version 25 using chi-squared tests and multivariate logistic regression analysis. Among Fort McMurray residents, 249 accessed the online survey, while 186 ultimately completed it, yielding a survey completion rate of 74.7%. Of these, 159 (85%) were females. After controlling for other variables in the regression model, respondents who expressed a desire to receive mental health counselling were more than seven times more likely to report suicidal ideation and thoughts of self-harm compared to the respondents who did not desire to receive mental health counselling (OR: 7.29; 95% CI: 1.19–44.58). Similarly, respondents who reported having abused alcohol in the past year were nearly four times more likely to report suicidal ideation or thoughts of self-harm compared to the respondents who said they had not abused alcohol in the past year (OR: 3.91; 95% CI: 1.05–14.57). A high prevalence of suicidal thoughts and thoughts of self-harm were reported among female residents of Fort McMurray. Timely access to adequate mental health support should be offered to female residents of communities impacted by multiple natural disasters, particularly residents who self-report alcohol abuse or desire to receive mental health counselling.

## 1. Introduction

Globally, 703,000 people die by suicide each year, according to the World Health Organization (WHO), and many more people attempt suicide [1]. Self-harm, suicidal behaviour, and suicide are major public health issues and the leading cause of injury and death [1,2,3,4]. Self-harm is a significant risk factor for suicide [5]. Self-harm and suicide are highly associated with genetic vulnerability and psychiatric, psychological, familial, social, and cultural factors [4]. Similarly, suicidal behaviour is considered the result of complicated interactions of psychological, cultural and social factors that have impacted an individual. Still, none of these factors is solely responsible for suicide [6]. Suicidal behaviour can also be defined as an action by which individuals hurt themselves (self-aggression), no matter the extent of lethal intent and the acknowledgement of the genuine reason for this action [7,8]. In broader terms, suicidal behaviour can be conceptualized as a range: beginning with thoughts of self-destruction, extending to threats, suicidal gestures, suicide attempts and ultimately, suicide [7,8]. Suicide can sometimes be an impulsive phenomenon. Therefore, it is argued that if support is provided in the instant of impulsivity, ideally at the time of suicidal ideation or thought, the crisis may be neutralized [9]. Published literature indicates self-harm is a bodily response to affect regulation and can be explained as a dysfunctional competency [10]. According to Jeong et al., previous suicide attempts were a significant risk factor for completed suicide [11]. This study also reported that among those with suicidal ideation, 3.9% had attempted suicide, and specifically in women, those with the lowest income level were associated with higher rates of attempts. The Centers for Disease Control and Prevention USA reports that in 2020, an estimated 12.2 million adults seriously thought about suicide, 3.2 million made a plan, and 1.2 million attempted suicide. This meant a 30% increase in suicide rates and nearly 46,000 deaths in 2020 [3]. In a meta-analysis and systematic review by Farooq et al., the prevalence of suicidal ideation reported in the general population was 11.5%, which is higher than the prevalence reported in the literature [12]. Another meta-analysis and systematic review also reported a global prevalence of approximately 9.8% for suicidal thoughts in the past 12 months [13]. A study by Botega in Brazil registered a lifetime prevalence rate of 17.1% for suicidal ideation, 4.8% for plans and 2.8% for suicide attempts. This study also noted that suicidal ideation was more frequently reported by women, particularly those living alone and individuals with mental disorders [8]. However, a cross-sectional survey reported higher rates: 23.2% of the respondents had thought about suicide, and 28.3% had made a suicide attempt in 2008 [14]. One study reported a cross-national lifetime prevalence of suicidal ideation, plans, and attempts at 9.2% and a notable 60% transition from ideation to plan and attempt within the first year after ideation onset across all countries [15]. Over the past decade, there has been a rise in the treatment of suicidal individuals, yet this has not been reflected in the incidence rates of suicidal behaviour, which have remained mostly the same [2].

Published literature indicates that factors such as gender and mental illness are highly correlated with suicide, and women are more prone to suicidal ideation. At the same time, men are more likely to attempt suicide [16]. One study also confirmed several risk factors for suicidal ideation, including being female, younger, less educated, single or unmarried, and having a mental disorder [15,17], with unmarried females reporting suicidal ideation more often [17]. Another study said that the common general significant predictor of suicidal ideation was anxiety and depression, whilst females showed numerous specific significant predictors, including educational threat [18]. Suicidal ideation is also significantly more endorsed among females than male adolescents, and females are more likely to adopt self-destructive behaviour or self-harm than males [18]. The reported prevalence estimate of recent suicidal thoughts in women was higher than in men [17]. Nonfatal suicidal behaviours are reported to be more prevalent among women and individuals who are unmarried or those with a psychiatric disorder [2], and females were hospitalized for attempted suicide at about 1.5 times the rate of males [19]. However, another study in China found no significant difference between males and females concerning suicidal ideation [17]. A study reported that married people had a lower suicide rate than single, divorced or widowed [9,20]. Thus, marriage is suggested to be protective for males in terms of suicidal ideation and suicide risk but not necessarily for females [9]. One study reported that anxiety, alcohol abuse, and illicit drug use were independently related to ideation outcomes, both suicidal ideation with a plan and without a plan [14]. Drinking was also reported to be a significant factor in male suicidal ideation but showed no difference for females [17]. Published literature indicates that alcohol use concerning self-harm, history of prior psychiatric treatment in particular of self-harm, and employment problems became more prevalent in 2008 [21]. Reports also indicate that individuals who have died by suicide often have a diagnosis of alcohol abuse and alcohol dependence [9].

In Canada, suicide remains the second most important cause of death of persons between 15 and 34 years of age, and suicide mortality in Alberta, Quebec, and New Brunswick continues to increase [22]. Another study in Canada reported that a 1% increase in the unemployment rate increases the suicide rate by 2.1%. In Alberta, however, a 1% increase in the unemployment rate resulted in a 2.8% increase in the suicide rate per 100,000 Albertans, which was considerably higher than the national average [6]. Results from the Canadian Community Health Survey–Mental Health (2012 CCHS) indicated that the lifetime prevalence of moderate depression in 2012 was 11.3%, the annual prevalence was 4.7% (95% CI 4.3% to 5.1%) in 2012, and the percentage of participants with lifetime moderate depressive episodes (MDEs) reporting a past year episode was 41.9% (95% CI 39.1% to 44.8%) [23]. In Alberta, among the general population, research during the COVID-19 pandemic recorded a 6-week prevalence for moderate or high stress of 85.6%, with anxiety at 47.0% and depression symptoms at 44.0%.

Fort McMurray, a northern Alberta, Canada city, had experienced its economic downturn even before the pandemic. From the onset of 2014, crude prices began to slide downward, with the price of oil dropping to below USD 27 a barrel in February 2016. This resulted in layoffs in the oil sector, with thousands of workers being laid off in one day in 2015 [24]. The residents of Fort McMurray have also encountered many disasters in the last five years, including the 2016 wildfire, the 2020 floods, and the ongoing COVID-19 pandemic. These traumatic experiences are believed to harm survivors’ mental health and general well-being [25,26]. 

A recent study in Fort McMurray reported that participants who experienced COVID-19, flooding, and wildfire traumas were four times more likely to have likely MDD, eleven times likely to have probable PTSD and eighteen times more likely to express GAD symptoms in comparison to the respondents who experienced COVID-19-only trauma [27]. Exposure to trauma increases the likelihood of PTSD, MDD and GAD, which invariably may increase the risk of suicidal ideation. A study in Canada also reported that among participants with past-year MDD, generalized anxiety disorder was present in 24.9%, and suicide attempts were reported by 6.6% of the participants with past-year MDD [28].

In Fort McMurray, approximately 100 businesses were estimated to have been destroyed in the 2020 flooding, and even more than a year later, less than 50 have re-opened [24]. The ongoing pandemic has also led to the decline and loss of businesses and business activity across virtually all industries, challenging the mental stability of impacted individuals [29,30]. Individuals who survive multiple traumatic events, such as wildfire and flooding, often experience prolonged and short-term problems that affect their lives [31]. It can also lead to immediate and perpetual mental health complications such as depression, anxiety, and post-traumatic stress disorder (PTSD) [32,33,34,35]. The published literature reports a high correlation between suicidal ideation and self-harm with increased mental health burden, unemployment, age and female gender [2,9,20,22]. Considering those mentioned above, and with exposure to several traumas, females in Fort McMurray are expected to have increased self-harm tendencies and suicidal thoughts or ideation. To date, no previous study has examined the prevalence and potential correlates of any community globally impacted by wildfires, flooding, and the COVID-19 pandemic. Thus, the goal of this study was to examine the prevalence and potential sociodemographic, clinical, and related correlates of thoughts of self-harm and suicidal ideation among the female residents of Fort McMurray. 

## 2. Methodology

### 2.1. Study Setting

This study was conducted in Fort McMurray (FMM), Alberta, Canada. Fort McMurray is the urban service area of Northern Alberta, with a total population of 111,687, according to the 2018 census [36]. The municipality has a young population; males and females constitute 54.9% and 45.1%, respectively. Regarding age distribution, almost half of the population lies between the ages of 20 and 44, whereas the 30–34 age group represents the majority and accounts for 12.3% of the total population. Seniors (65 years of age and over) account for only 2.8% of the population [37]. Many inhabitants are employed in the nearby oil sands [37,38].

### 2.2. Study Design

This cross-sectional study used an online self-administered questionnaire survey distributed via REDCap [39] to the general adult residents in Fort McMurray. This was facilitated with the help of social media platforms of community partners, including the public and Catholic school boards, Keyano College, the Canadian Mental Health Association and the Alberta Building Trades Association. No incentives were offered to the respondents. 

### 2.3. Ethical Approval

The study received ethical approval from the Health Research Ethics Board of the University of Alberta (Pro00066054). Informed consent was implied when respondents completed and returned the survey responses.

### 2.4. Data Collection and Used Measures

Data collection occurred from adult residents of Fort McMurray between 24 April and 2 June 2021. We planned to exclude data from any respondents younger than 18. The survey questionnaire took about 5–10 min to complete, and it included a blend of questions that assessed sociodemographic information, including age, marital and employment status, and data related to the severity of mental health conditions, including depression, anxiety, resilience, and post-traumatic disorder (PTSD). Additionally, the survey questions included the history of receiving psychotropic medications, including antidepressants and sleeping tablets, and the history of misuse of drugs such as alcohol abuse and marijuana use or abuse. The survey questions were adapted from previously used surveys in related studies that gathered data on the mental health conditions among the residents of Fort Mc Murray [33,40,41]. Data from this study represent all responses received from the survey respondents who self-identified as females.

The principal clinical outcome of this study was suicidal ideation and thoughts of self-harm among females. The variable was assessed using the validated screening scale of the Patient Health Questionnaire-9 (PHQ-9) [42]. The scale’s ninth question was “Bothered by thoughts that you would be better off dead, or of hurting yourself in the last two weeks”. The question initially had four responses (Not at all, several days, more than half the days, and nearly every day), and for analysis, this was collapsed into only two group responses, those who have had passive death wishes/thoughts of self-harm in the last two weeks, and those who have not had passive death wish/thoughts of self-harm in the previous two weeks (PHQ-9 ≥ 1 and PHQ-9 < 1), respectively. Respondents selecting responses other than zero were classified as having suicidal ideation and thoughts of self-harm. Clinical variables used in the analysis included the Generalized Anxiety Disorder 7-item (GAD-7) Scale (GAD-7 score ≥ 10 indicates likely GAD) [43], the Patient Health Questionnaire-9 (PHQ-9; a score ≥ 10 indicates likely MDD) [42], the Post-Traumatic Stress Disorder (PTSD) Checklist–Civilian Version (PCL-C) scale (PCL-C score of ≥44 indicates likely PTSD) [44,45], and the Brief Resilience Scale (BRS) (BRS average score < 3 indicates perceived low resilience) [46]. The scales were studied as categorical variables for prevalence estimates. 

### 2.5. Statistical Analysis

Results were analyzed using SPSS Version 25 [47]. We studied the prevalence of the primary study outcome, suicidal ideation and thoughts of self-harm, and its correlates among sociodemographic and clinical factors, including mental health conditions, drugs, and psychotropic medications. Association analysis was applied using chi-squared and Fisher’s exact tests with two-tailed significance (*p* ≤ 0.05). Logistic regression analysis was employed to identify the significant predictors of developing suicidal ideation and thoughts of self-harm while controlling for other factors in the model. The variables that showed either significance (*p* ≤ 0.05) or near significance (0.1 ≥ *p* > 0.05) obtained from the univariate chi-squared analysis were ascertained to be included in the logistic regression analysis. The dependent factor was the dichotomous variable of the suicidal ideation and thoughts of self-harm question. Adjusted odds ratios (AOR) and confidence intervals were used to determine the effect of the respondents’ sociodemographic and clinical factors on self-report of likely suicidal ideation and thoughts of self-harm. Before the regression analysis, a correlational analysis was performed to exclude any strong intercorrelations (Spearman’s correlation coefficient (r_s_) of 0.7 to 1.0 or −0.7 to −1.0) among predictor variables. There was no missing data imputation, and the reported data represents the complete responses.

### 2.6. Sample Size Estimation 

With adult females representing 39.5% of a total population of 111,687, according to the 2018 census [36], a 95% confidence and a ±5% margin of error, using an online script (https://www.surveymonkey.com/mp/sample-size-calculator/, accessed on 10 February 2022), the sample size needed for prevalence estimates for suicidal ideation and thoughts of self-harm among female residents of Fort McMurray is 385.

## 3. Results

Among FM residents, 249 accessed the online survey, while 186 ultimately completed it, yielding a response rate of 74.7%. Of those 186, 159 (85%) were females. The results are reported for this subgroup of respondents. A total of 79 (49.7%) of the respondents were 40 years of age or less, while 80 (50.3%) were above 40. The age range was 18 to 71 years, with a mean of 49.15 years and a standard deviation of 11.14 years. Table 1 summarizes the sociodemographic and clinical characteristics concerning the respondents’ age groups. Most female respondents were employed (151, 95%) and in a relationship (115, 72.3%). About one in three (52, 32.7%) was on antidepressants, and one in ten (17, 10.7%) was on sleeping tablets. A total of 63 (39.6%) of the respondents reported receiving mental health counselling in the past year, while 89 (56%) reported their desire to receive it. Regarding the drug abuse questions, 43 (27%) of the respondents self-reported alcohol abuse, while 18 (11.3%) self-reported their abuse of marijuana. 

Concerning the mental health conditions among the respondents, 67 (45.9%) presented with moderate to severe depression symptoms, 63 (43.8%) presented with moderate to severe anxiety symptoms, 58 (40.8%) presented with likely PTSD symptoms, and 58 (39.5%) presented with low resilience symptoms. Overall, 26/146 (17.8%) respondents reported having had passive death wishes or thoughts of self-harm in the last two weeks before completing the survey.

Table 2 presents the association analysis of the demographic and clinically related variables with the suicidal thought variable. Except for age, which did not show a statistically significant association (*p* = 0.82), and the use/abuse of marijuana, which showed a near significant association (*p* = 0.097), all other variables showed a statistically significant association with suicidal ideation and thoughts of self-harm among females (*p* < 0.05). 

Table 2 shows that unemployed participants (57.1%), as well as those not in a relationship (28.2%), those currently taking antidepressants (31.3%) or sleeping tablets (43.8%), those who have received mental health counselling (28.6%) or would like to receive mental health counselling (29.3%), those who have abused alcohol in the past year (38.9%), those who self-reported moderate-to-severe depression symptoms (31.3%), moderate-to-severe anxiety symptoms (30.2%), or PTSD symptoms (31%), or those with low resilience (25.9%) were more likely to report suicidal ideation and thoughts of self-harm.

### Multivariate Logistic Regression Analysis

In Table 3, we included the 11 variables that showed significance and the one that showed a near-significant association with suicidal ideation and thoughts of self-harm in the logistic regression model. No high correlation was observed among the predictors (r_s_ < 0.7). The model was statistically significant; *Χ*^2^ (df = 12; n = 142) = 39.27, *p* < 0.00, accounting for 24.2% (Cox and Snell R^2^) to 39.9% (Nagelkerke R^2^) of the variance, and correctly classified 86.6% of the cases. 

Table 3 demonstrates the summary results of the logistic regression analyses regarding the predictive effect of sociodemographic and clinically related variables on suicidal ideation and thoughts of self-harm. From the table, the desire to receive mental health counselling and self-reported alcohol abuse were the only two significant predictors for suicidal ideation and thoughts of self-harm within the female study cohort after controlling for the other variables in the model. Respondents who reported their desire to receive mental health counselling were more than seven times as likely to report suicidal ideation and thoughts of self-harm (Wald = 4.63) compared to the respondents who reported no desire to receive mental health counselling while controlling for other model variables (OR: 7.29; 95% CI: 1.19–44.58).

Similarly, respondents who reported having abused alcohol in the past year were nearly four times more likely to express suicidal ideation and thoughts of self-harm compared to the respondents who reported non-alcohol abuse in the past year while controlling for other model variables (OR: 3.91; 95% CI: 1.05–14.57).

## 4. Discussion

This study found the prevalence of passive death wishes or thoughts of self-harm in the past two weeks among female residents of Fort McMurray was 17.8%. This prevalence was much higher compared to a pooled global prevalence of approximately 9.8% for suicidal thoughts in the past 12 months reported in a meta-analysis and systematic review [13] and a prevalence of 11.5% for suicidal ideation recorded among the general population in another meta-analysis [12]. A study conducted among female college students in India reported a prevalence of 12.5% for suicidal ideation [48]. Several studies suggest that exposure to a natural disaster such as wildfire and flooding can lead to both immediate and long-term mental disorders such as anxiety, depression and PTSD in affected individuals [32,33,34,35] and that these mental disorders are risks factors for suicidal thoughts and self-harm [15,17]. This may contribute to the increased likelihood of suicidal thoughts or thoughts of self-harm in our study sample.

This study has also established that the desire to receive mental health counselling and alcohol abuse in the past year are the only two out of the twelve variables that predict suicidal ideation and thoughts of self-harm among residents of Fort McMurray when all other variables are controlled for in the logistic regression model. In this study, demographic variables, including employment and marital status and clinical variables, such as the presence of moderate to high anxiety, moderate to high depression, likely PTSD and low resilience, did not independently predict suicidal ideation and thoughts of self-harm among residents of Fort McMurray. This finding contrasts with other literature that reported a positive and independent association between suicidal ideation or self-harm and increased mental health burden, unemployment and age [2,9,20,22]. In particular, being in a relationship and marriage has been reported to be protective against suicidal ideation [9,20]. However, in this study, relationship status did not independently predict suicidal ideation or thoughts of self-harm. This is contrary to a study suggesting that unmarried females are more likely to report suicidal ideation [17] and nonfatal suicidal behaviours [2] than married females. 

In this study, only 5% of participants were unemployed, and unemployment did not independently predict suicidal ideation or thoughts of self-harm. Other studies have suggested that unemployment is a risk factor for suicidal ideation, self-harm or self-destruction and suicide [9,49,50]. 

Respondents who expressed a desire to receive mental health counselling were more than seven times as likely to report suicidal ideation and thoughts of self-harm compared to respondents who reported no desire to receive mental health counselling. Female residents of Fort McMurray who are part of the general population have suffered several traumatic events in the last five years, including the wildfire of 2016, the COVID-19 pandemic, and the flooding of 2020, which may account for the high prevalence of moderate to high depression symptoms (45.9%), moderate to high anxiety symptoms (43.8%), likely PTSD (40.8%) and low resilience (39.5%) recorded in this study. One study reported several risk factors for suicidal ideation, including having a mental or psychiatric disorder or difficulties [15,17], such as depression [51]. A study by Patten also reported that suicide attempts were reported by 6.6% of the participants with past-year MDD [28]. 

Furthermore, a systematic review also reported that the main risk factors for suicidal ideation include mental health difficulties [12]. Although the mental health conditions themselves did not independently predict the presence of suicidal ideation in this study, the desire to receive mental health counselling was counter-intuitive and, in part, contrary to the published literature [15,17]. A possible explanation for this finding is that we used self-reported screening tools to determine the presence or absence of likely mental disorders in this study. Although the tools are all validated, it may be necessary for a future study to ask participants to specify in particular what they desire to seek mental health services for. 

Another possible explanation for why sociodemographic and clinical factors did not independently predict suicidal ideation or thoughts of self-harm in respondents in this study, but the desire to receive mental health counselling predicted suicidal ideation or thoughts of self-harm in respondents, is that the desire to receive mental health counselling may be a better indicator of the presence of mental distress than the individual clinical variables. This explanation is plausible because individuals who desire mental health counselling could have a combination of psychosocial stressors and mental health issues [33,35,40,52]. Thus, it is reasonable to conclude that although the individual sociodemographic and clinical variables were not independently predictive of suicidal ideation or thoughts of self-harm, a combination of sociodemographic and clinical factors may be predictive.

This study has shown that 27% of the respondents self-reported alcohol abuse, while 11.3% reported cannabis abuse. Respondents who reported having abused alcohol in the past year were nearly four times more likely to express suicidal ideation and thoughts of self-harm than those who reported no alcohol abuse in the past year. Our study is in line with an early study that reported that alcohol abuse is independently associated with suicidal ideation with and without a plan or self-harm [14]. Self-harm has also been associated with alcohol use [21], and alcohol abuse has been identified as one of the common precursors of suicidal thoughts and self-harm [21]. A study in Canada reported that among participants with past-year MDD, 4.8% had past-year alcohol abuse, and 4.5% had alcohol dependence [28]. This study shows a relationship between MDD and alcohol abuse, both of which are risk factors for suicidal ideation and self-harm and hence explain the high prevalence recorded in our study. However, another study reported that alcohol abuse is a significant risk factor in male suicidal ideation but not in females [17]. In this study, cannabis abuse did not independently predict suicidal ideation or thoughts of self-harm, in contrast with studies that reported that substance abuse was linked to a high rate of suicide attempts [14]. 

### Limitations of the Study

This study is not without its limitations. First, the online survey was distributed through online social media platforms of community organizations in Fort McMurray, so not all residents might have had access to the survey leading to selection bias. Therefore, the data from this study represent a convenient sample of female residents and may not represent the adult female population of Fort McMurray. Thus, the study findings need to be interpreted with caution, as the sample may best be reflective of the residents of FMM who can be reached randomly through our intermediaries. Another potential bias is that those who responded could very likely be more of those who had felt the need for psychological support, which may affect the prevalence estimates for the desire to be dead/experienced thoughts of self-harm. In addition, it is impossible to specify how many individuals received the survey links. Hence, the response rate, calculated using the number of unique individuals who clicked on the survey link as the denominator rather than the number who received the link, might have been overestimated. Again, because male respondents to this study were few (27 males versus 159 females), the original focus of the study and the planned analysis was shifted from the general adult residents to only female participants. Thus, it was impossible to examine “gender” as a variable of interest in this study.

Furthermore, given that the sample size for female participants in the study was only 159 rather than the projected 385, the margin of error for our prevalence estimate for suicidal thoughts/thoughts of self-harm and other mental conditions was calculated using a confidence interval of 95% and an online script (https://www.surveymonkey.com/mp/margin-of-error-calculator/, accessed on 10 February 2022) was ±8% rather than the projected ±5%. Furthermore, the relatively small size may not represent the females in Fort McMurray. Again, demographic variables in the study may not reflect the demographics of female residents of Fort McMurray. Therefore, the study findings may not be generalizable to all female residents of Fort McMurray. Additionally, the ninth question on the PHQ-9 scale, which was used in assessing suicide risk, assesses two constructs simultaneously rather than independently assessing each contract. It is, therefore, uncertain if respondents respond in the affirmative to either the thought of being better off dead, thoughts of hurting themselves, or both. Finally, alcohol abuse was assessed for all participants using self-reports with the following question: “Have you abused alcohol in the past year?” Alcohol abuse was not pre-defined for study participants. Thus, each participant was left on their own to interpret what alcohol abuse meant to them, which has the potential for misinterpretation and inaccurate reporting of participants who met the criteria for alcohol abuse. 

Despite these limitations, our study provides important insights and evidence on the mental health effects, the role of alcohol abuse, unemployment and being married on the incidence of suicidal ideation or thoughts of self-harm among Fort McMurray females, which would be of interest to policymakers in planning mental health support for the community. 

## 5. Conclusions 

Suicidal ideation and thoughts of self-harm may advance to fatal suicide, so it is essential to reduce their incidence among the female population in Fort McMurray. To the best of the authors’ knowledge, this study is the first to establish an independent association between the desire to receive mental health counselling and suicidal thoughts or thoughts of self-harm. It is also the first study to validate the established association between problematic alcohol use and suicidal thoughts or thoughts of self-harm in female residents of Fort McMurray. In these regards, this study adds to the existing literature. The outcome of this study will help policymakers plan public-health strategies and healthcare policies to be tailored to help the Fort McMurray community mitigate the risk associated with suicidal ideation and self-harm. Individuals with suicidal ideation need timely support, which should be provided at the moment of impulsivity, to offset the crisis of probable suicide. Mobile text messages can provide an easily accessible and readily available resource to help reduce suicidal ideation and self-harm tendencies and is easily scalable and cost-effective. It can be applied at the population level as interventions such as supportive text messaging programs [53,54,55,56,57,58,59] and has been established to reduce stress, anxiety and depression in subscribers of the Text4Hope program in Alberta, Canada, during the COVID-19 pandemic [60,61,62,63,64]. In three randomized control trials, a supportive text message helped reduce problematic alcohol use [53,65,66], which can be implemented to help reduce alcohol intake. There is also a need for increased addiction counselling services to support this vulnerable population. Furthermore, individuals seeking mental health counselling should also be screened for alcohol use disorder and provided with alcohol counselling when indicated. 

## Figures and Tables

**Table 1:** Age distribution of sociodemographic and clinical characteristics.

Variables	≤40 Years n (%)	>40 Years n (%)	Total n (%)
**Employment status** Employed Unemployed	75 (94.9) 4 (5.1)	76 (95.0) 4 (5.0)	151 (95.0) 8 (5.0)
**Marital status** In a relationship Not in a relationship	59 (74.7) 20 (25.3)	56 (70.0) 24 (30.0)	115 (72.3) 44 (27.7)
**Are you currently on antidepressants for mental health concerns?** No Yes	54 (68.4) 25 (31.6)	53 (66.3) 27 (33.8)	107 (67.3) 52 (32.7)
**Are you currently on sleeping tablets for mental health concerns?** No Yes	71 (89.9) 8 (10.1)	71 (88.8) 9 (11.3)	142 (89.3) 17 (10.7)
**Have you received mental health counselling in the past year?** No Yes	41 (51.9) 38 (48.1)	55 (68.8) 25 (31.3)	96 (60.4) 63 (39.6)
**Would you like to receive mental health counselling?** No Yes	28 (35.4) 51 (64.6)	42 (52.5) 38 (47.5)	70 (44.0) 89 (56.0)
**Have you abused alcohol in the past year?** No Yes	58 (73.4) 21 (26.6)	58 (72.5) 22 (27.5)	116 (73.0) 43 (27.0)
**Have you used/abused marijuana in the past year?** No Yes	69 (87.3) 10 (12.7)	72 (90.0) 8 (10.0)	141 (88.7) 18 (11.3)
**Moderate to severe depression symptoms**	36 (51.4)	31 (40.8)	67 (45.9)
**Moderate to severe anxiety symptoms**	34 (49.3)	29 (38.7)	63 (43.8)
**Likely PTSD**	28 (41.2)	30 (40.5)	58 (40.8)
**Low resilience**	34 (48.6)	24 (31.2)	58 (39.5)
**Have had passive death wishes or thoughts of self-harm in the last two weeks** No Yes	57 (81.4) 13 (18.6)	63 (82.9) 13 (17.1)	120 (82.2) 26 (17.8)

**Table 2:** Chi-squared association between the demographic and clinical variables and the likelihood of experiencing passive death wishes or thoughts of self-harm in the preceding two weeks.

Variables	Have not had Passive Death Wish/Thoughts of Self-Harm in Last Two Weeks N (%)	Have had Passive Death Wishes/Thoughts of Self-harm in the Last Two Weeks N (%)	Chi^2^	*p*-Value
**Age category** ≤40y >40y	57 (81.4) 63 (82.9)	13 (18.6) 13 (17.1)	0.05	0.82
**Employment status** Employed Unemployed	117 (84.2) 3 (42.9)	22 (15.8) 4 (57.1)	7.77	0.01
**Marital status** In a relationship Not in a relationship	92 (86.0) 28 (71.8)	15 (14.0) 11 (28.2)	3.93	0.047
**Are you currently on antidepressants for a mental health concern?** No yes	87 (88.8) 33 (68.8)	11 (11.2) 15 (31.3)	8.83	<0.01
**Are you currently on sleeping tablets for a mental health concern?** No yes	111 (85.4) 9 (56.3)	19 (14.6) 7 (43.8)	8.26	<0.01
**Have you received mental health counselling in the past year?** No Yes	80 (88.9) 40 (71.4)	10 (11.1) 16 (28.6)	7.19	<0.01
**Would you like to receive mental health counselling?** No Yes	62 (96.9) 58 (70.7)	2 (3.1) 24 (29.3)	16.79	<0.01
**Have you abused alcohol in the past year?** No Yes	98 (89.1) 22 (61.1)	12 (10.9) 14 (38.9)	14.51	<0.01
**Have you used/abused marijuana in the past year?** No Yes	110 (84.0) 10 (66.7)	21 (16.0) 5 (33.3)	2.75	0.097
**Depression** At most mild depression Moderate to severe depression	74 (93.7) 46 (68.7)	5 (6.3) 21 (31.3)	15.5	<0.01
**Anxiety** At most low anxiety Moderate to severe anxiety	75 (92.6) 44 (69.8)	6 (7.4) 19 (30.2)	12.79	<0.01
**PTSD** Not likely PTSD Likely PTSD	77 (91.7) 40 (69.0)	7 (8.3) 18 (31.0)	12.19	<0.01
**Resilience** High to normal resilience Low resilience	77 (87.5) 43 (74.1)	11 (12.5) 15 (25.9)	4.26	0.04

**Table 3:** Logistic regression model for respondents’ likelihood to present with passive death wishes or thoughts of self-harm.

	Coefficient	SE	Wald	df	*p*-Value	AOR	95% CI for AOR	
							Lower	Upper
**Employment status** Not employed	−0.040	1.009	0.002	1	0.968	0.961	0.133	6.938
**Marital status** In a relationship Not in a relationship	0.540	0.616	0.770	1	0.380	1.717	0.514	5.740
**Are you currently on antidepressants for a mental health concern?** No yes	0.477	0.602	0.627	1	0.428	1.611	0.495	5.248
**Are you currently on sleeping tablets for a mental health concern?** No yes	0.800	0.830	0.928	1	0.335	2.225	.437	11.330
**Have you received mental health counselling in the past year?** No Yes	0.022	0.630	0.001	1	0.972	1.022	0.297	3.512
**Would you like to receive mental health counselling?** No Yes	1.987	0.924	4.628	1	0.031 *	7.293	1.193	44.575
**Have you abused alcohol in the past year?** No Yes	1.362	0.672	4.109	1	0.043 *	3.905	1.046	14.573
**Have you used/abused marijuana in the past year?** No Yes	−0.478	0.928	0.266	1	0.606	0.620	0.101	3.820
**Likely depression** At most mild depression Moderate to severe depression	1.099	0.665	2.731	1	0.098	3.002	0.815	11.061
**Likely anxiety** At most low anxiety Moderate to severe anxiety	0.753	0.656	1.318	1	0.251	2.123	0.587	7.680
**Likely PTSD** Not likely PTSD Likely PTSD	0.268	0.654	0.168	1	0.682	1.307	0.363	4.707
**Resilience** High to normal resilience Low resilience	−0.527	0.620	0.724	1	0.395	0.590	0.175	1.989
Constant	−4.977	0.997	24.941	1	0.000	0.007		

* Significance at *p* < 0.05. AOR: adjusted odds ratio. CI: confidence interval. SE: standard error. df: degrees of freedom.

## Data Availability

Data for this study is available on reasonable request from the corresponding author.
